# ITGB1 Contributes to Matrix Stiffness–Induced Lipid Raft Organization and Promotes Hepatocellular Carcinoma Cell Migration

**DOI:** 10.3390/ijms27219577

**Published:** 2026-10-26

**Authors:** Xu Zhang, Xinyu Liu, Yan Liu, Guanbin Song

**Affiliations:** 1 Key Laboratory of Biorheological Science & Technology, Ministry of Education, College of Bioengineering, Chongqing University, Chongqing 400030, China; 202419021021@stu.cqu.edu.cn; 2 School of Life Science, Southwest University, Chongqing 400715, China; lxy20021029@email.swu.edu.cn

**Keywords:** hepatocellular carcinoma, matrix stiffness, integrin β1, lipid rafts, cell migration

## Abstract

Matrix stiffening is a characteristic pathological feature of the hepatocellular carcinoma (HCC) microenvironment and promotes HCC cell migration. Lipid rafts are cholesterol–enriched plasma membrane microdomains involved in cell adhesion and migration. However, whether matrix stiffness promotes lipid raft organization and whether lipid rafts contribute to stiffness–promoted HCC cell migration remain unclear. Using two HCC cell lines cultured on polyacrylamide hydrogels with distinct stiffnesses, we found that stiff matrix enhanced cell migration, increased Area-normalized GM1 fluorescence, and upregulated Caveolin-1 and Flotillin-1 protein expression, consistent with enhanced lipid raft–associated membrane organization. Cholesterol depletion with methyl-β–cyclodextrin (MβCD) reduced GM1 fluorescence and attenuated migration on the Stiff matrix. Cholesterol replenishment partially restored GM1 fluorescence and wound closure after MβCD treatment. To explore how extracellular matrix stiffness is coupled to lipid raft organization, we next examined integrin β1 (ITGB1), a major extracellular matrix mechanoreceptor. The Stiff matrix increased ITGB1 protein expression, ITGB1–GM1 colocalization, and ITGB1 levels in raft-enriched fractions. ITGB1 knockdown reduced GM1 fluorescence, decreased Caveolin-1 and Flotillin-1 levels in raft-enriched fractions, and suppressed migration under Stiff–matrix conditions. These findings support a contributory role for ITGB1 in stiffness-associated lipid raft organization and HCC cell migration.

## 1. Introduction

Hepatocellular carcinoma (HCC), the predominant form of primary liver cancer, remains a major cause of cancer–related mortality worldwide [1]. HCC frequently arises in the setting of chronic liver injury, fibrosis, or cirrhosis, where excessive extracellular matrix (ECM) deposition, remodeling, and crosslinking progressively increase tissue stiffness [2,3,4,5,6]. Increased matrix stiffness acts as an important mechanical cue that promotes HCC cell migration and invasion through diverse mechanotransduction mechanisms [7,8,9]. However, the membrane-level mechanisms linking matrix stiffness to HCC cell migration remain poorly understood.

One membrane-level process potentially involved in this mechanical response is the dynamic organization of lipid rafts, nanoscale membrane domains enriched in cholesterol, and sphingolipids [10,11,12,13]. By transiently concentrating selected receptors and signaling molecules, lipid rafts can compartmentalize signal transduction at the plasma membrane [14]. Lipid rafts are involved in cell adhesion, cytoskeletal regulation, migration, and other physiological and pathological processes, and their dysregulation has been associated with cancer progression [15,16,17]. In non-HCC cells, matrix stiffness has been linked to changes in Caveolin-1 and β1 integrin–associated membrane responses, while disruption of cholesterol–rich membrane domains affects Stiff–matrix–induced cellular behaviors [18,19]. However, whether matrix stiffness promotes lipid raft organization in HCC cells and whether lipid rafts contribute to stiffness–promoted HCC cell migration remain unclear.

Integrin β1 (ITGB1) is a transmembrane β subunit that pairs with multiple α subunits to form heterodimeric integrin receptors involved in cell–ECM adhesion and mechanosensing [20]. Previous studies have established ITGB1 as an important mediator of stiffness sensing in HCC. Early work showed that β1–integrin and focal adhesion kinase contribute to stiffness-dependent HCC cell responses, including proliferation [21]. Subsequent studies demonstrated that increased matrix stiffness elevates ITGB1 expression and promotes HCC proliferation, invasion, and migration through ITGB1–dependent signaling and cytoskeletal regulation [8,9,22]. However, whether ITGB1-dependent mechanosensing is coupled to changes in plasma–membrane lipid raft organization in HCC remains largely unexplored. In other cellular contexts, ITGB1 has been reported to associate dynamically with lipid rafts. IGF–I induces rapid Caveolin-1–dependent recruitment of ITGB1 to lipid rafts [23], whereas enhanced incorporation of ITGB1 into lipid rafts has been linked to increased cell migration in osteoblastic and osteosarcoma cells [24]. More broadly, integrin-mediated adhesion can alter membrane order and membrane–domain organization [25,26]. Conversely, Caveolin-1 and cholesterol-rich membrane domains can regulate β1-integrin membrane localization and trafficking. Caveolin-1 depletion impairs β1-integrin recruitment or endocytosis in different cellular systems, whereas integrin-mediated adhesion can itself regulate Caveolin-1–dependent membrane–domain organization [19,23,27,28]. Together, these observations support a reciprocal relationship between β1 integrin and Cav1/raft-associated membrane organization rather than a strictly unidirectional pathway. These observations raise the possibility that ITGB1 could couple extracellular matrix stiffness to lipid raft organization, but whether this relationship operates in HCC cells under increased matrix stiffness remains unresolved.

Based on these observations, we hypothesized that ITGB1 serves as a mechanosensitive link between extracellular matrix stiffness and lipid raft organization, thereby contributing to HCC cell migration. In this study, we examined whether ITGB1 contributes to stiffness-associated changes in raft–related membrane readouts and HCC cell migration. Using stiffness-defined polyacrylamide hydrogels, cholesterol depletion, and ITGB1 knockdown approaches, we show that ITGB1 contributes to stiffness-induced lipid raft organization and HCC cell migration.

## 2. Results

### 2.1. Increased Matrix Stiffness Promotes HCC Cell Migration

**Figure 1:**
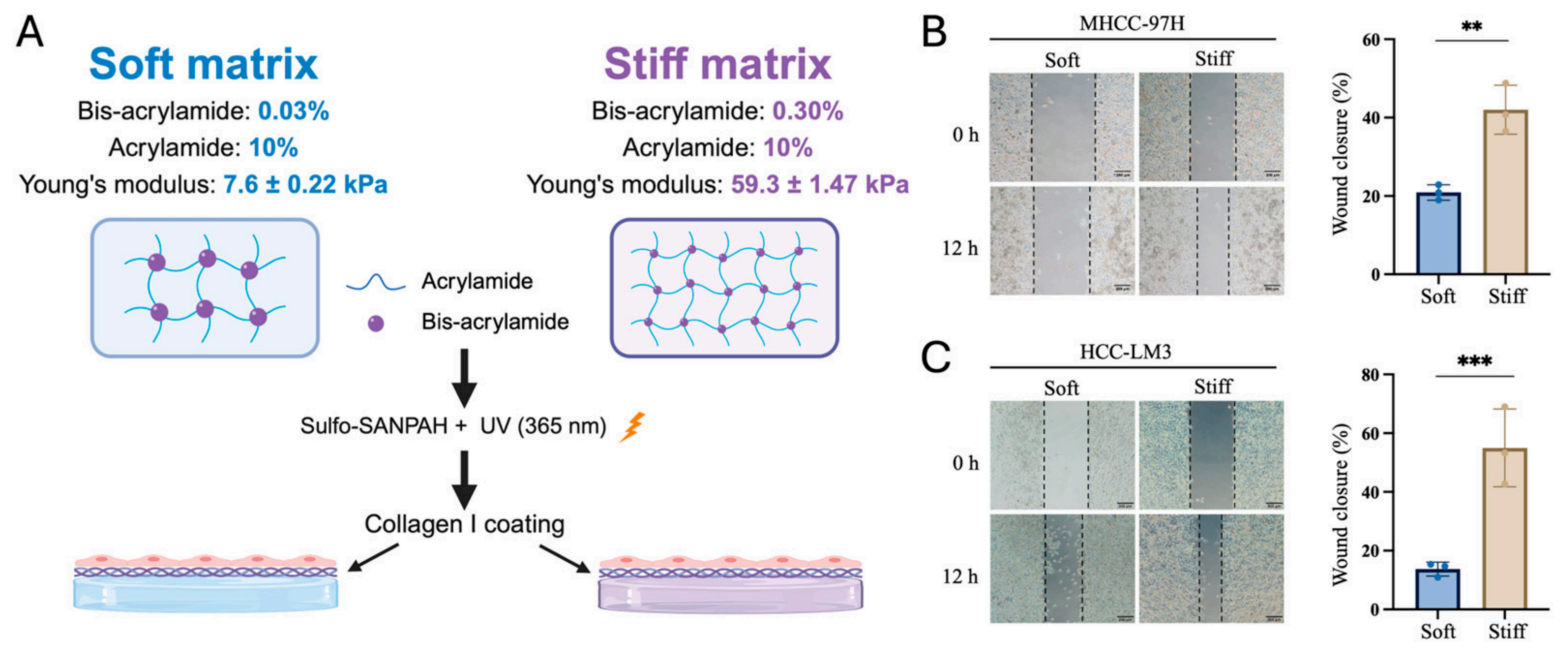
Establishment of stiffness-defined polyacrylamide hydrogel models and evaluation of matrix stiffness–dependent HCC cell migration. (**A**) Schematic illustration of the Soft and Stiff polyacrylamide hydrogel models used in this study. Soft and Stiff hydrogel substrates exhibited Young’s moduli of 7.6 ± 0.22 kPa and 59.3 ± 1.47 kPa, respectively, and were coated with collagen I before cell culture. Mechanical characterization was performed using six independent hydrogel samples per group. Created in BioRender (web-based version). Zhang, X. (2026). https://BioRender.com/jinhcui (accessed on 10 September 2026). (**B**,**C**) Representative wound-healing images and quantification of wound closure in MHCC-97H (**B**) and HCC-LM3 (**C**) cells cultured on Soft or Stiff matrix for 12 h. Scale bars, 200 μm. Migration experiments were performed using three independent biological replicates. Data are presented as mean ± SD. Statistical significance was determined using a two-tailed unpaired Student’s *t*–test. ** *p* < 0.01, *** *p* < 0.001.

To investigate the effect of matrix stiffness on HCC cell migration, we established collagen I–coated polyacrylamide (PA) hydrogel models with different stiffnesses. Based on previously reported liver stiffness values and stiffness-defined HCC models, two mechanically distinct PA hydrogel conditions were selected, with Young’s moduli of 7.6 ± 0.22 kPa and 59.3 ± 1.47 kPa, respectively [21,29]. In this study, these two conditions were referred to as “Soft” and “Stiff”, respectively (Figure 1A).

We next examined whether matrix stiffness influenced HCC cell migration using wound-healing assays. Compared with those cultured on the Soft matrix, both MHCC-97H and HCC-LM3 cells on the Stiff matrix exhibited significantly increased wound closure after 12 h (Figure 1B,C). These results showed greater wound closure on the Stiff matrix than on the Soft matrix in both HCC cell lines, providing an experimental model for subsequent investigation of the underlying molecular mechanisms.

### 2.2. Increased Matrix Stiffness Promotes Lipid Raft–Associated Membrane Organization in HCC Cells

**Figure 2:**
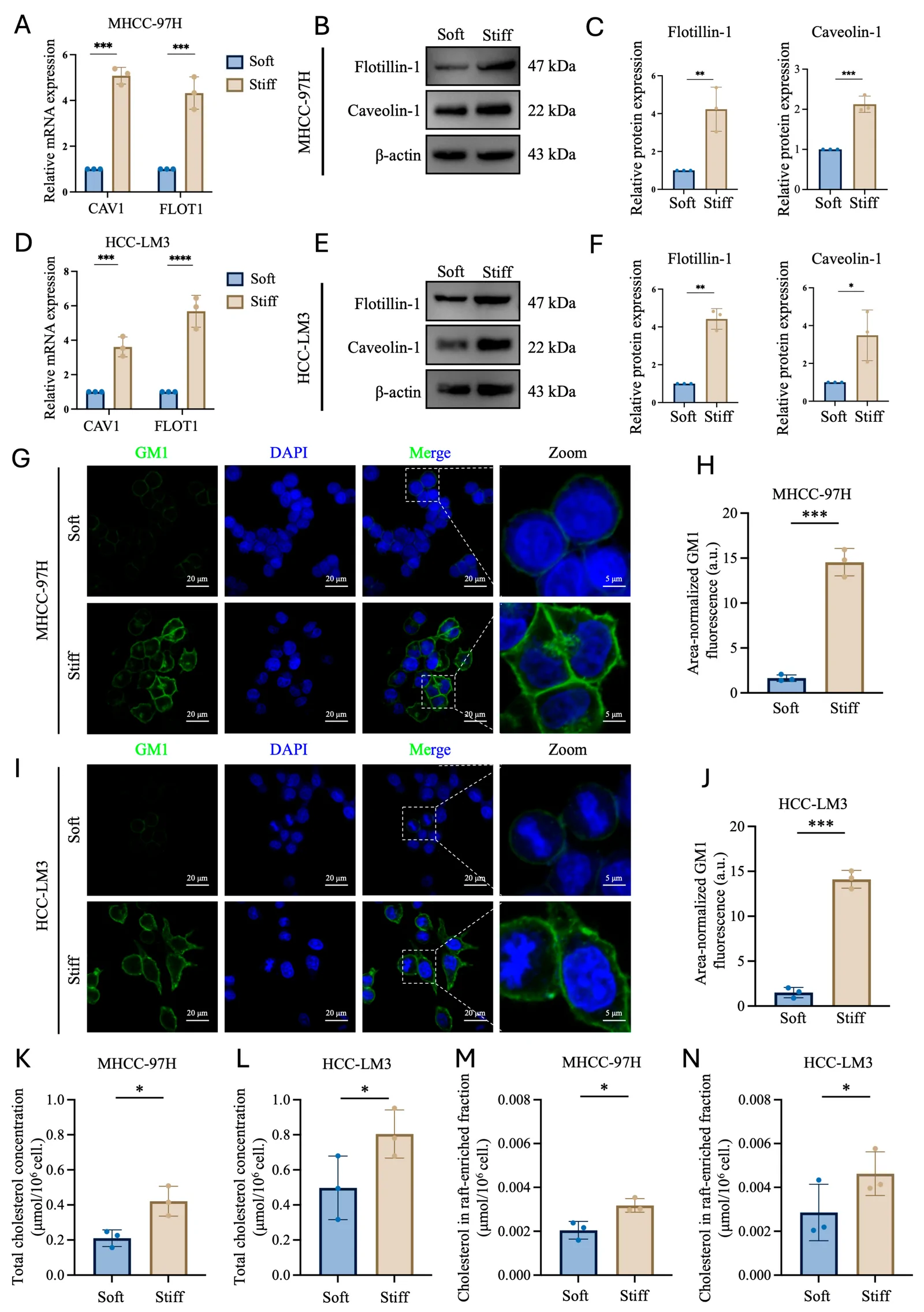
Increased matrix stiffness enhances lipid raft–associated features in HCC cells. (**A**,**D**) Relative *CAV1* and *FLOT1* mRNA levels in MHCC-97H (**A**) and HCC-LM3 (**D**) cells cultured on Soft or Stiff matrix, measured by RT-qPCR. (**B**,**E**) Representative immunoblots of Flotillin-1 and Caveolin-1 in MHCC-97H (**B**) and HCC-LM3 (**E**) cells; β-actin served as the loading control. (**C**,**F**) Densitometric quantification of Flotillin-1 and Caveolin-1 protein abundance normalized to β-actin in MHCC-97H (**C**) and HCC-LM3 (**F**) cells. (**G**,**I**) Representative confocal images of membrane GM1 detected by fluorescent cholera toxin B subunit (CTB; green) in MHCC-97H (**G**) and HCC-LM3 (**I**) cells. Nuclei were counterstained with DAPI (blue). Dashed boxes indicate the enlarged regions. Scale bars, 20 μm in overview images and 5 μm in enlarged images. (**H**,**J**) Quantification of area-normalized GM1 fluorescence in MHCC-97H (**H**) and HCC-LM3 (**J**) cells. Background-corrected integrated GM1 fluorescence was normalized to the projected area of individual cells. (**K**,**L**) Total cellular cholesterol concentrations in MHCC-97H (**K**) and HCC-LM3 (**L**) cells cultured on Soft or Stiff matrix. (**M**,**N**) Cholesterol concentrations in raft-enriched fractions isolated from MHCC-97H (**M**) and HCC-LM3 (**N**) cells cultured on Soft or Stiff matrix. Data are presented as mean ± SD from three independent experiments. Statistical significance was determined using a two-tailed unpaired Student’s *t*–test. * *p* < 0.05, ** *p* < 0.01, *** *p* < 0.001, and **** *p* < 0.0001.

To evaluate whether matrix stiffness influences lipid raft–associated membrane organization, we examined *CAV1* and *FLOT1* expression together with membrane GM1 fluorescence signals in two HCC cell lines. Caveolin-1 and Flotillin-1 are commonly used as lipid raft–associated markers [30]. RT-qPCR revealed significantly higher *CAV1* and *FLOT1* mRNA levels in MHCC-97H and HCC-LM3 cells cultured on the Stiff matrix than in those cultured on the Soft matrix (Figure 2A,D). Consistent with the transcript data, Caveolin-1 and Flotillin-1 protein expression levels were also increased on the Stiff matrix (Figure 2B,C,E,F).

We then visualized membrane GM1 using fluorescently labeled cholera toxin B subunit (CTB), which binds GM1 with high affinity [31]. Both cell lines displayed stronger GM1 fluorescence on the Stiff matrix than on the Soft matrix (Figure 2G,I). Quantification confirmed a significant increase in area-normalized GM1 fluorescence in MHCC-97H and HCC-LM3 cells on the Stiff matrix (Figure 2H,J). To further characterize the cholesterol–associated membrane changes, we quantified both total cellular cholesterol and cholesterol in the isolated raft-enriched fractions. Total cellular cholesterol was significantly increased in cells cultured on the Stiff matrix compared with the Soft matrix in both MHCC-97H and HCC-LM3 cells (Figure 2K,L). Cholesterol levels in the raft-enriched fractions were also significantly higher under the Stiff condition in both cell lines (Figure 2M,N).

Taken together, the coordinated increases in Caveolin-1 and Flotillin-1 expression, area-normalized GM1 fluorescence, and raft-associated cholesterol provide complementary evidence supporting enhanced lipid raft–associated membrane organization under increased matrix stiffness.

### 2.3. Cholesterol Depletion Attenuates Stiffness-Promoted HCC Cell Migration, Whereas Cholesterol Replenishment Partially Restores the Phenotype

**Figure 3:**
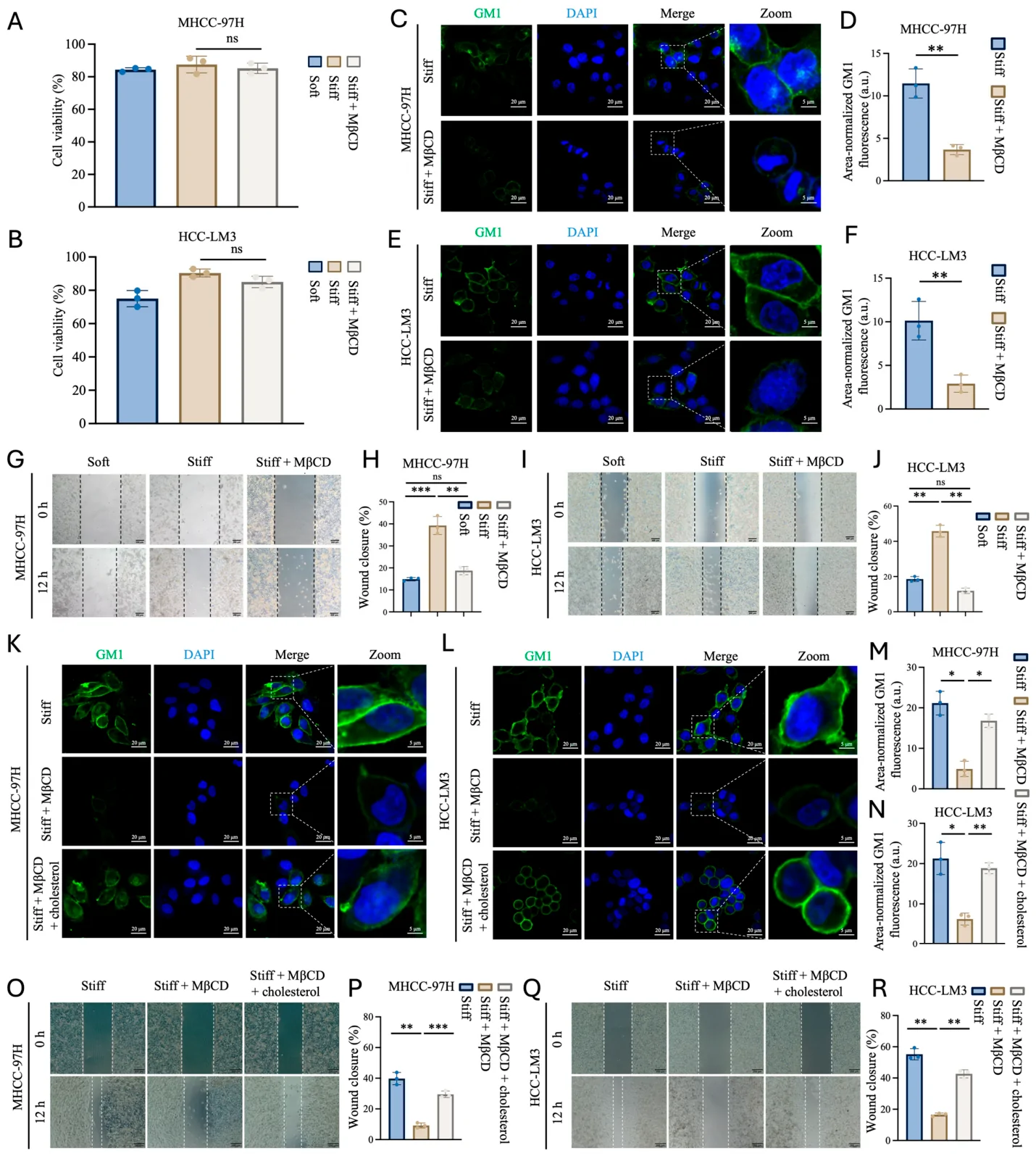
Cholesterol depletion attenuates stiffness-promoted HCC cell migration, and cholesterol replenishment partially restores the phenotype. (**A**,**B**) CCK-8–based cell viability of MHCC-97H (**A**) and HCC-LM3 (**B**) cells cultured on the Soft matrix, Stiff matrix, or Stiff matrix treated with MβCD. Cell viability was normalized to the parallel Control group cultured on standard tissue-culture-treated plates, which was defined as 100%. (**C**,**E**) Representative confocal images of membrane GM1 detected by fluorescent cholera toxin B subunit (CTB; green) in MHCC-97H (**C**) and HCC-LM3 (**E**) cells cultured on the Stiff matrix with or without MβCD treatment. Nuclei were counterstained with DAPI (blue). Dashed boxes indicate the enlarged regions. Scale bars, 20 µm in overview images and 5 µm in enlarged images. (**D**,**F**) Quantification of area-normalized GM1 fluorescence in MHCC-97H (**D**) and HCC-LM3 (**F**) cells. (**G**,**I**) Representative wound-healing images of MHCC-97H (**G**) and HCC-LM3 (**I**) cells cultured on the Soft matrix, Stiff matrix, or Stiff matrix treated with MβCD. For all groups, 0 h was defined after the 60 min MβCD treatment or time-matched control incubation and subsequent medium replacement. The same fields were imaged 12 h later. Scale bars, 200 µm. (**H**,**J**) Quantification of wound closure during the 12 h post–treatment observation period in MHCC-97H (**H**) and HCC-LM3 (**J**) cells. (**K**,**L**) Representative confocal images of membrane GM1 in MHCC-97H (**K**) and HCC-LM3 (**L**) cells under Stiff, Stiff + MβCD, and Stiff + MβCD + cholesterol conditions. Scale bars, 20 µm in overview images and 5 µm in enlarged images. (**M**,**N**) Quantification of area-normalized GM1 fluorescence in MHCC-97H (**M**) and HCC-LM3 (**N**) cells following cholesterol replenishment. (**O**,**Q**) Representative wound-healing images of MHCC-97H (**O**) and HCC-LM3 (**Q**) cells under Stiff, Stiff + MβCD, and Stiff + MβCD + cholesterol conditions. For all groups, 0 h was defined after completion of the 60 min depletion stage, the subsequent 4 h replenishment or time-matched control stage, and medium replacement. The same fields were imaged 12 h later. Scale bars, 200 µm. (**P**,**R**) Quantification of wound closure during the 12 h observation period following completion of the replenishment or time-matched control stage in MHCC-97H (**P**) and HCC-LM3 (**R**) cells. Data are presented as mean ± SD from three independent experiments. Statistical significance for GM1 fluorescence in panels D and F was determined using a two-tailed unpaired Student’s *t*–test. Data in panels (**A**,**B**,**H**,**J**,**M**,**N**,**P**,**R**) were analyzed using one-way ANOVA followed by Tukey’s multiple-comparisons test. * *p* < 0.05, ** *p* < 0.01, *** *p* < 0.001; ns, not significant.

To determine whether membrane cholesterol contributes to stiffness-promoted HCC cell migration, we depleted membrane cholesterol using methyl-β-cyclodextrin (MβCD). MβCD has been widely used to perturb lipid raft structure by extracting membrane cholesterol [19,32,33]. Based on previous studies, cells cultured on the Stiff matrix were treated with 5 mM MβCD for 60 min before subsequent analyses. To assess whether acute MβCD treatment affected cell viability, CCK-8 assays were performed. No significant difference in CCK-8–based cell viability was detected between the Stiff and Stiff + MβCD groups in either MHCC-97H or HCC-LM3 cells (Figure 3A,B).

Following MβCD treatment, CTB staining revealed markedly reduced Area-normalized GM1 fluorescence in both MHCC-97H and HCC-LM3 cells cultured on the Stiff matrix (Figure 3C,E). Quantification after normalization to individual cell projected area confirmed significantly lower GM1 fluorescence in the Stiff + MβCD group than in the Stiff group (Figure 3D,F). Consistently, MβCD treatment significantly reduced wound closure during the subsequent 12 h observation period in both HCC cell lines on the Stiff matrix (Figure 3G–J).

To further determine whether these effects were cholesterol dependent and reversible, we performed cholesterol add-back experiments following MβCD treatment. Cholesterol replenishment using an MβCD–cholesterol complex has been previously used to restore cellular cholesterol levels after MβCD-mediated cholesterol depletion [34]. In both HCC cell lines, cholesterol replenishment significantly restored area-normalized GM1 fluorescence compared with the Stiff + MβCD group, although the recovery remained partial relative to the Stiff group (Figure 3K–N). Cholesterol replenishment significantly increased wound closure relative to the Stiff + MβCD group during the subsequent 12 h observation period in both HCC cell lines (Figure 3O–R).

Together, these results show that cholesterol depletion disrupts lipid raft–associated membrane organization and suppresses HCC cell migration under Stiff-matrix conditions, whereas cholesterol replenishment partially restores both GM1 fluorescence and migration. These findings further support a cholesterol-dependent contribution of lipid raft–associated membrane organization to stiffness-promoted HCC cell migration.

### 2.4. Increased Matrix Stiffness Enhances ITGB1 Association with Lipid Rafts in HCC Cells

**Figure 4:**
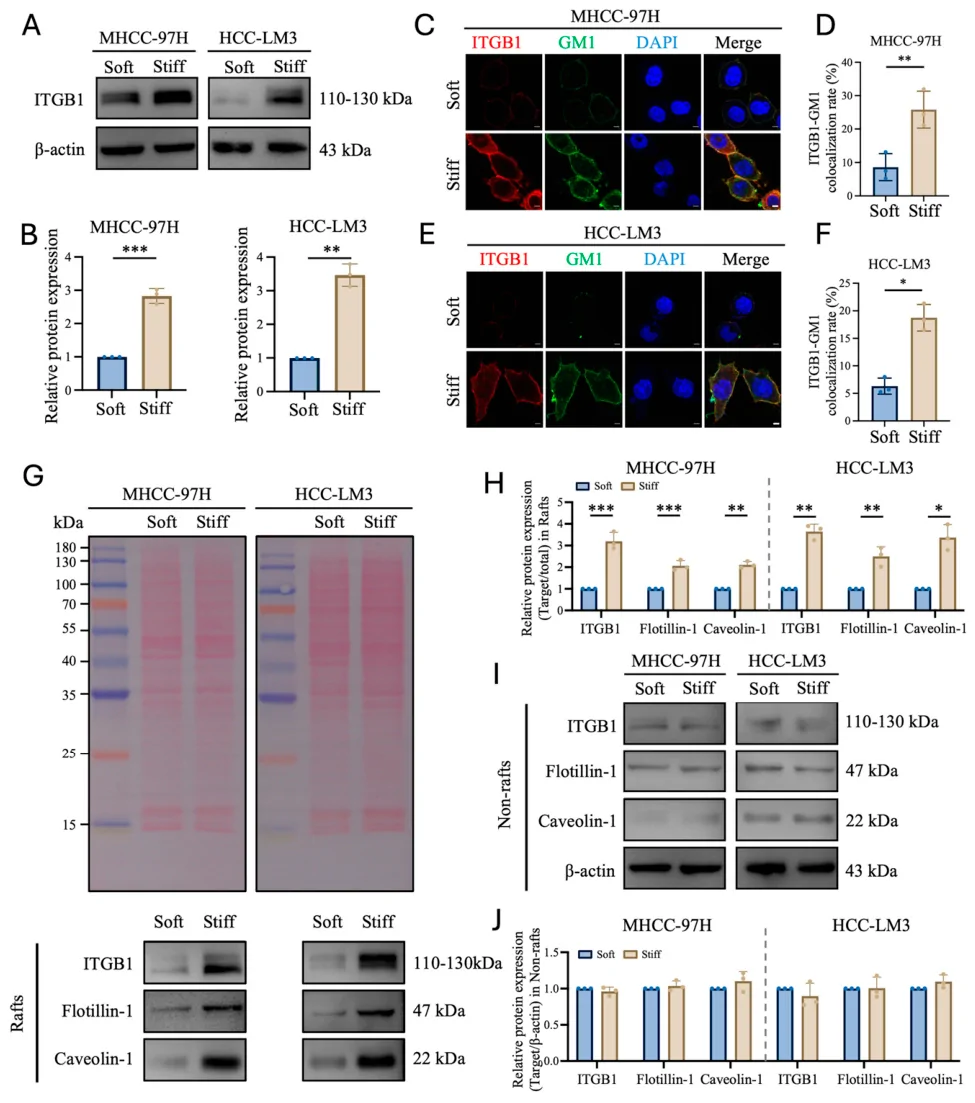
Increased matrix stiffness enhances ITGB1 association with lipid rafts in HCC cells. (**A**) Representative immunoblot of ITGB1 in total-cell lysates from MHCC-97H and HCC-LM3 cells cultured on Soft or Stiff matrix; β-actin served as the loading control. (**B**) Densitometric quantification of ITGB1 abundance normalized to β-actin in MHCC-97H and HCC-LM3 cells. (**C**,**E**) Representative immunofluorescence images of ITGB1 (red), GM1 (green), and nuclei (DAPI; blue) in MHCC-97H (**C**) and HCC-LM3 (**E**) cells cultured on Soft or Stiff matrix. Scale bars, 5 μm. (**D**,**F**) Quantification of ITGB1-GM1 colocalization in MHCC-97H (**D**) and HCC-LM3 (**F**) cells. (**G**) Ponceau S total-protein staining and representative immunoblots of ITGB1, Flotillin-1, and Caveolin-1 in detergent-resistant, raft-enriched fractions from cells cultured on Soft or Stiff matrix. (**H**) Quantification of proteins in raft-enriched fractions, normalized to the corresponding Ponceau S total-protein signal and then to the Soft matrix group. (**I**) Representative immunoblots of ITGB1, Flotillin-1, and Caveolin-1 in detergent-soluble non-raft fractions; β-actin served as the loading control. (**J**) Quantification of proteins in non-raft fractions normalized to β-actin and the corresponding Soft matrix group. Data are mean ± SD from *n* = 3 independent experiments. Two-tailed unpaired Student’s *t*-test was used for Soft-versus-Stiff comparisons. * *p* < 0.05, ** *p* < 0.01, *** *p* < 0.001.

Integrin β1 (ITGB1) serves as an important mediator of extracellular matrix adhesion and mechanical sensing [20]. We therefore examined whether matrix stiffness affected ITGB1 abundance in HCC cells. Western blot analysis showed that ITGB1 protein levels were significantly increased in both MHCC-97H and HCC-LM3 cells cultured on the Stiff matrix compared with those cultured on the Soft matrix (Figure 4A,B). The two closely migrating ITGB1 bands observed in the immunoblots are consistent with previously reported glycosylation-dependent forms of β1 integrin, including a partially glycosylated precursor and a more fully glycosylated mature form [35].

We next investigated whether the stiffness-induced increase in ITGB1 was associated with GM1-positive membrane regions. Immunofluorescence analysis revealed enhanced ITGB1 signals and increased ITGB1-GM1 colocalization rates in cells cultured on the Stiff matrix in both HCC cell lines (Figure 4C–F), suggesting enhanced association between ITGB1 and lipid raft–associated membrane domains under Stiff-matrix conditions.

To further examine the membrane distribution of ITGB1, raft-enriched and non-raft fractions were isolated and analyzed by Western blotting. Compared with the Soft matrix, the Stiff matrix significantly increased ITGB1 abundance in raft-enriched fractions in both MHCC-97H and HCC-LM3 cells (Figure 4G,H). The abundance of the raft-associated proteins Flotillin-1 and Caveolin-1 was also increased in raft-enriched fractions under Stiff matrix conditions. In contrast, ITGB1, Flotillin-1, and Caveolin-1 abundance in non-raft fractions showed no significant changes or only limited alterations (Figure 4I,J). These findings show increased ITGB1 abundance in raft-enriched fractions under Stiff-matrix conditions. They do not distinguish increased total abundance from altered partitioning between membrane fractions.

### 2.5. ITGB1 Knockdown Attenuates Stiffness-Induced Lipid Raft–Associated Changes

**Figure 5:**
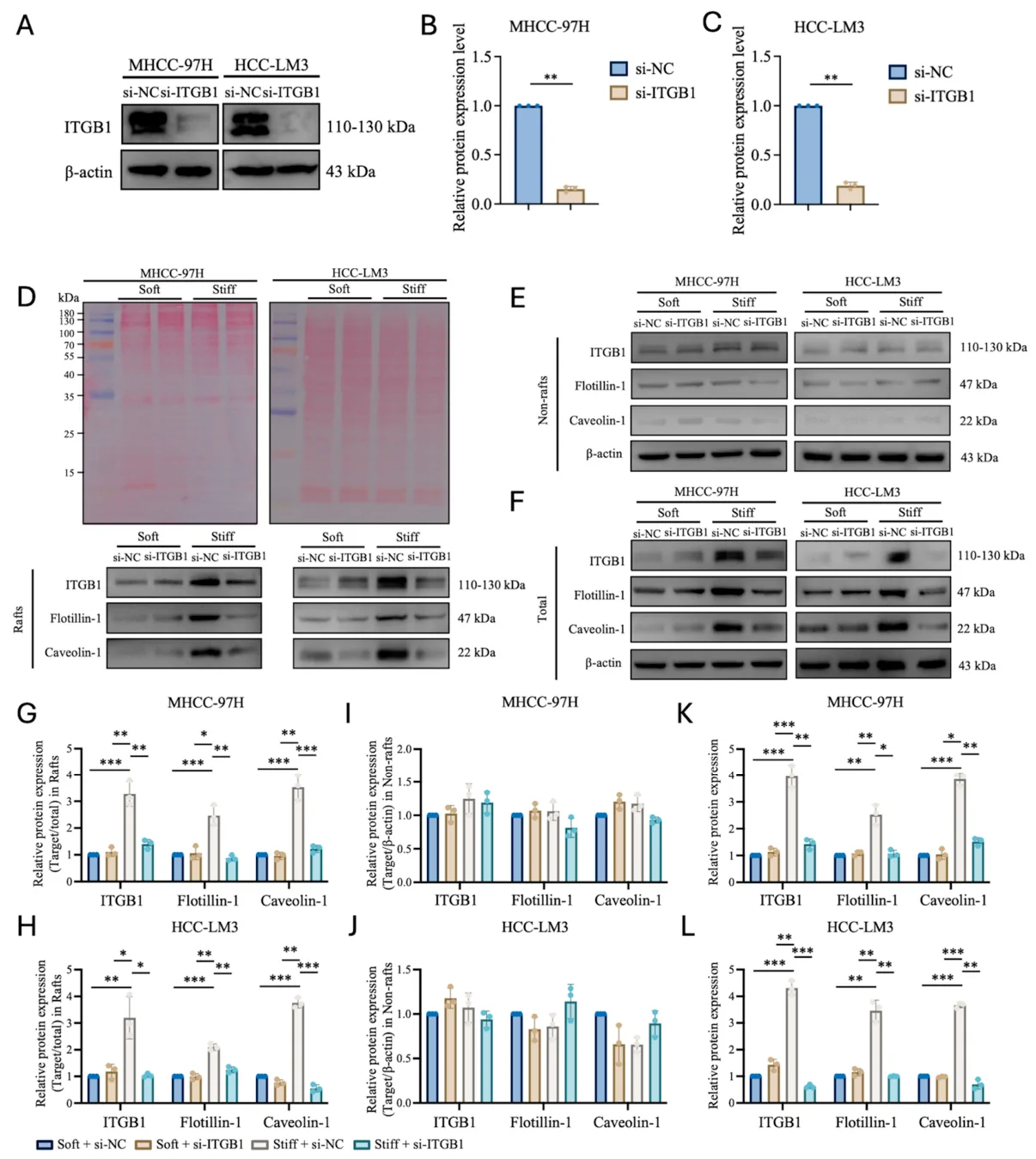
ITGB1 knockdown attenuates stiffness-induced increases in raft-associated proteins. (**A**) Representative immunoblot confirming ITGB1 knockdown in MHCC-97H and HCC-LM3 cells transfected with ITGB1-targeting siRNA (si-ITGB1) or negative-control siRNA (si-NC); β-actin served as the loading control. (**B**,**C**) Quantification of ITGB1 protein levels in MHCC-97H (**B**) and HCC-LM3 (**C**) cells. (**D**–**F**) Representative immunoblots of ITGB1, Flotillin-1, and Caveolin-1 in raft-enriched (**D**), non-raft (**E**), and total-cell (**F**) fractions from the indicated groups. Ponceau S staining is shown for raft-enriched fractions, whereas β-actin served as the loading control for non-raft and total-cell fractions. (**G**–**L**) Quantification of the corresponding proteins in raft-enriched (**G**,**H**), non-raft (**I**,**J**), and total-cell (**K**,**L**) fractions from MHCC-97H and HCC-LM3 cells. Raft-enriched fractions were normalized to Ponceau S total-protein signals, whereas non-raft and total-cell fractions were normalized to β-actin. Data are presented as mean ± SD from three independent experiments. A two-tailed unpaired Student’s *t*–test was used for (**B**,**C**), and two-way ANOVA followed by Tukey’s multiple-comparisons test was used for (**G**–**L**). * *p* < 0.05, ** *p* < 0.01, *** *p* < 0.001.

To further determine whether ITGB1 contributes to matrix stiffness–induced lipid raft changes, we first knocked down ITGB1 in MHCC-97H and HCC-LM3 cells using siRNA. Western blot analysis showed markedly reduced ITGB1 protein levels in the si-ITGB1 groups compared with the corresponding si-NC groups in both cell lines, confirming effective ITGB1 knockdown (Figure 5A–C).

The si-NC and si-ITGB1–treated cells were subsequently cultured on the Soft or Stiff matrix, and ITGB1, Flotillin-1, and Caveolin-1 were analyzed in raft-enriched fractions, non-raft fractions, and total-cell lysates. In raft-enriched fractions, Stiff + si-NC cells showed higher levels of all three proteins than Soft + si-NC cells, whereas ITGB1 knockdown markedly attenuated these stiffness-induced increases in both MHCC-97H and HCC-LM3 cells (Figure 5D,G,H). By contrast, ITGB1 knockdown caused limited changes in the corresponding non-raft fractions, where the abundance of these proteins remained relatively unchanged across conditions (Figure 5E,I,J). Total-cell lysates also showed reduced levels of all three proteins following ITGB1 knockdown (Figure 5F,K,L). Together, these findings support a role for ITGB1 in stiffness-induced increases in raft-associated proteins, with comparatively limited changes in the corresponding non-raft fractions.

### 2.6. ITGB1 Knockdown Attenuates Stiffness-Associated Lipid Raft Organization and HCC Cell Migration

**Figure 6:**
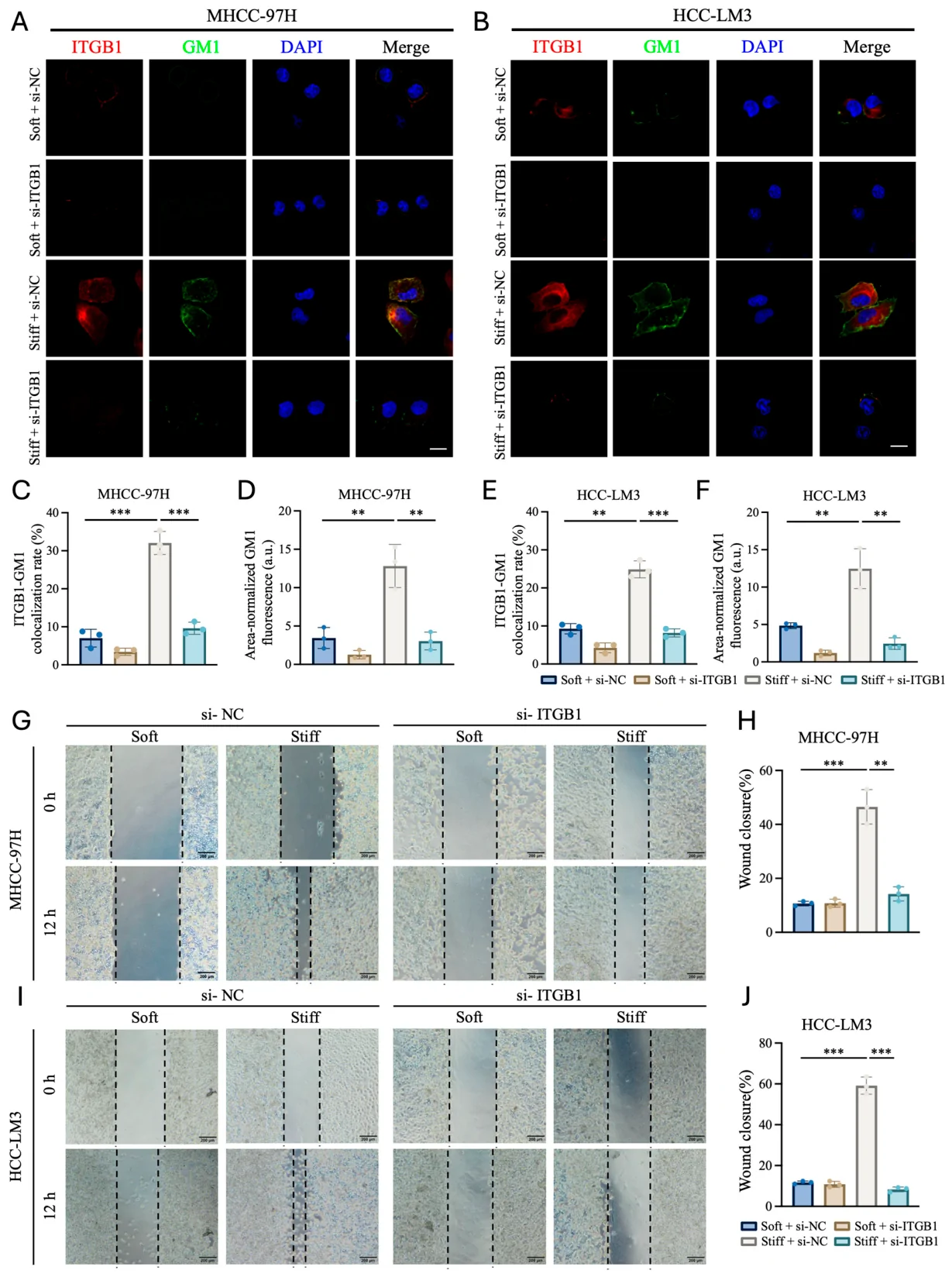
ITGB1 knockdown reduces stiffness-associated lipid raft organization and HCC cell migration. (**A**,**B**) Representative confocal images of ITGB1 (red), GM1 (green), and nuclei (DAPI; blue) in si-NC– or si-ITGB1–transfected MHCC-97H (**A**) and HCC-LM3 (**B**) cells cultured on Soft or Stiff matrix. Scale bars, 10 μm. (**C**–**F**) Quantification of ITGB1–GM1 colocalization rate (**C**,**E**) and area-normalized GM1 fluorescence (**D**,**F**) in MHCC-97H (**C**,**D**) and HCC-LM3 (**E**,**F**) cells. (**G**,**I**) Representative wound-healing images of si-NC– or si-ITGB1–transfected MHCC-97H (**G**) and HCC-LM3 (**I**) cells cultured on Soft or Stiff matrix at 0 and 12 h. Dashed lines indicate wound boundaries; scale bars, 200 μm. (**H**,**J**) Quantification of wound closure in MHCC-97H (**H**) and HCC-LM3 (**J**) cells. Data are presented as mean ± SD from three independent experiments. Statistical significance was determined using two-way ANOVA followed by Tukey’s multiple-comparisons test. For clarity, only comparisons between Soft + si-NC and Stiff + si-NC and between Stiff + si-NC and Stiff + si-ITGB1 are indicated. ** *p* < 0.01, *** *p* < 0.001.

To examine the contribution of ITGB1 to stiffness-associated membrane changes, si-NC– or si-ITGB1–transfected cells were cultured on Soft or Stiff matrices. ITGB1–GM1 colocalization and area-normalized GM1 fluorescence were then quantified. In si-NC–transfected cells, the Stiff matrix significantly increased ITGB1–GM1 colocalization and area-normalized GM1 fluorescence compared with the Soft matrix. Under Stiff-matrix conditions, ITGB1 knockdown significantly reduced both ITGB1–GM1 colocalization and Area-normalized GM1 fluorescence in MHCC-97H and HCC-LM3 cells (Figure 6A–F). The reduction in GM1 fluorescence was consistent with the decreased Flotillin-1 and Caveolin-1 levels in raft-enriched fractions observed in Figure 5.

Wound-healing assays were then used to examine the effect of ITGB1 knockdown on cell migration. In si-NC–transfected cells, the Stiff matrix significantly increased wound closure compared with the Soft matrix. ITGB1 knockdown significantly reduced wound closure under Stiff=matrix conditions in both MHCC-97H and HCC-LM3 cells (Figure 6G–J).

Together with the raft-fractionation results in Figure 5, these findings support a role for ITGB1 in stiffness-associated lipid raft organization and enhanced HCC cell migration.

## 3. Discussion

This study investigated a membrane-level mechanism through which matrix stiffness promotes HCC cell migration. Compared with the Soft matrix, the Stiff matrix significantly enhanced the migration of MHCC-97H and HCC-LM3 cells and increased Area-normalized GM1 fluorescence together with Caveolin-1 and Flotillin-1 expression, supporting increased lipid raft organization. MβCD-mediated membrane cholesterol depletion reduced GM1 fluorescence and migration under Stiff-matrix conditions, indicating that lipid raft integrity contributes to the migratory response induced by matrix stiffness. We further found that the Stiff matrix increased total ITGB1 expression, ITGB1–GM1 colocalization, and ITGB1 abundance in raft-enriched fractions. Conversely, ITGB1 knockdown reduced Area-normalized GM1 fluorescence, decreased Caveolin-1 and Flotillin-1 levels in raft-enriched fractions, and suppressed migration under Stiff-matrix conditions. Together, these findings support a role for ITGB1 in matrix stiffness–induced lipid raft organization and link this membrane response to enhanced HCC cell migration.

Lipid rafts are dynamic membrane microdomains whose organization, composition, and protein distribution can be regulated by membrane lipid composition and cellular signaling states [13]. Much of the existing work has focused on biochemical or metabolic regulation of lipid rafts. For example, in HCC cells, inhibition of DHCR24-mediated cholesterol biosynthesis reduces CTB-labeled lipid rafts and suppresses cell migration and invasion [33]. More recently, Qiu et al. showed that ACSL4-mediated membrane phospholipid remodeling alters the localization and interaction of ITGB1 and CD47 within lipid rafts, indicating that membrane lipid metabolic state can regulate protein organization in raft domains [36]. In contrast, studies directly addressing whether extracellular mechanical cues regulate lipid raft organization remain relatively limited. Integrin-mediated adhesion has been shown to alter plasma membrane order [25], while Yeh et al. reported that matrix stiffness regulates Caveolin-1 and β1 integrin and that perturbation of cholesterol-dependent membrane domains affects cell spreading and focal adhesion formation on the Stiff matrix [19]. Buwa et al. further showed that increasing matrix stiffness promotes Caveolin-1 Tyr14 phosphorylation [18]. However, these studies mainly focused on membrane order, Caveolin-1/ITGB1 regulation, or focal adhesion–related responses and did not directly establish whether matrix stiffness promotes lipid raft organization in HCC cells. In the present study, the Stiff matrix consistently increased GM1 fluorescence together with Caveolin-1 and Flotillin-1 expression in two HCC cell lines, providing further evidence that lipid raft organization is responsive to changes in the mechanical properties of the ECM. Together with previous studies on biochemical and metabolic regulation, our findings support the view that lipid rafts can respond not only to changes in membrane lipid composition and biochemical state but also to extracellular mechanical cues and may therefore represent a potential membrane-level interface where biochemical and mechanical information converge.

In HCC, previous studies have shown that increased matrix stiffness enhances β1-integrin–dependent signaling and malignant behaviors, including proliferation, invasion, invadopodia formation, and migration [8,9,22]. These findings establish ITGB1 as an important component of HCC mechanotransduction but have largely focused on downstream focal-adhesion signaling and cytoskeletal regulation rather than membrane–domain organization. ITGB1 is a key transmembrane component of cell–ECM adhesions and mechanosensing, and its association with lipid rafts can be dynamically regulated [20,23,24]. IGF-I induces rapid Caveolin-1–dependent recruitment of ITGB1 to lipid rafts [23], while BMP-2 increases the incorporation of ITGB1 into lipid rafts in association with enhanced migration of osteoblastic and osteosarcoma cells [24]. These findings indicate that ITGB1 distribution within the plasma membrane is responsive to extracellular signals. More recently, Qiu et al. showed that ACSL4 knockdown reduced ITGB1 levels in raft fractions of TNBC cells without significantly altering ITGB1 abundance in whole-cell lysates, whereas supplementation with polyunsaturated phospholipids restored raft-associated ITGB1. Notably, GM1 staining was largely unchanged, suggesting that membrane phospholipid remodeling in this model mainly affected ITGB1 localization within lipid rafts rather than lipid raft organization itself [36]. In the present study, the Stiff matrix increased total ITGB1 expression, ITGB1–GM1 colocalization, and ITGB1 abundance in raft-enriched fractions, whereas ITGB1 levels in non-raft fractions showed no significant change. Thus, although our findings are consistent with previous evidence that ITGB1 is dynamically associated with lipid rafts, the response to matrix stiffness differs from that induced by membrane phospholipid remodeling. Because total ITGB1 abundance also increased on the Stiff matrix, the higher ITGB1 level in raft-enriched fractions cannot be explained simply by selective translocation from non-raft to raft regions. Rather, matrix stiffness may increase overall ITGB1 abundance while also enhancing its association with raft-related membrane regions. The relative contribution of these two processes remains to be determined and raises a further question of whether ITGB1 only associates with lipid rafts in response to extracellular cues or also participates in regulating lipid raft organization. The present study did not directly assess the activation state of ITGB1; therefore, whether matrix stiffness also regulates ITGB1 activation independently of changes in its abundance or membrane association remains to be determined.

ITGB1 knockdown experiments further suggest that the relationship between ITGB1 and lipid rafts may not be limited to the localization of ITGB1 within pre-existing raft domains. Under Stiff-matrix conditions, ITGB1 knockdown reduced ITGB1–GM1 colocalization, decreased Area-normalized GM1 fluorescence, and lowered Caveolin-1 and Flotillin-1 levels in raft-enriched fractions, whereas the corresponding changes in non-raft fractions were comparatively limited. The concordant changes in membrane lipid and raft-associated protein readouts support the possibility that ITGB1 is not merely recruited to lipid rafts but also contributes to stiffness-induced lipid raft organization. However, because an siRNA-resistant ITGB1 rescue experiment was not performed, potential sequence-specific off-target effects of the siRNA cannot be completely excluded, and the knockdown results should therefore be interpreted as supporting rather than definitively establishing an ITGB1-dependent mechanism. Previous studies provide a basis for such a relationship. Gaus et al. showed that integrin-mediated adhesion regulates plasma membrane order, with cell detachment causing a rapid loss of membrane order together with internalization and increased mobility of CTB-labeled membrane domains [25]. Yeh et al. further reported reciprocal regulation between β1 integrin and Caveolin-1 under different matrix stiffness conditions, showing that β1 integrin activation or clustering could attenuate the reduction in Caveolin-1 induced by a soft matrix, whereas Caveolin-1 and cholesterol-dependent membrane domains were also required to maintain β1 integrin levels and focal adhesion formation [19]. These findings suggest a bidirectional relationship in which lipid rafts provide a membrane environment for ITGB1 localization and function, while ITGB1-mediated adhesion and mechanotransduction may in turn influence raft-associated membrane organization. Our findings are consistent with a possible reciprocal relationship between ITGB1 and lipid raft–associated membrane organization in stiffness-exposed HCC cells. However, the present experiments do not establish bidirectional regulation, and the directionality and molecular basis of this relationship remain unresolved.

Lipid rafts have been implicated in tumor-cell migration and invasion by organizing receptors, adhesion molecules, and downstream signaling components at the plasma membrane [16,37]. In HCC, lipid raft-associated TLR7 signaling promotes cell migration [38], whereas inhibition of DHCR24-mediated cholesterol synthesis reduces lipid raft formation together with HCC cell migration and invasion [33]. Similar effects have been reported in other tumor types; for example, ACSL4-mediated membrane phospholipid remodeling promotes ITGB1 localization and activation within lipid rafts, leading to FAK activation and enhanced TNBC invasion and metastasis [36]. Consistent with these studies, the stiffness-induced increase in HCC cell migration in our study was accompanied by increased GM1 fluorescence and elevated Caveolin-1 and Flotillin-1 levels. Disruption of cholesterol-dependent raft structure by MβCD reduced both GM1 fluorescence and migration under Stiff-matrix conditions. In parallel, ITGB1 knockdown decreased GM1 fluorescence and raft-associated Caveolin-1 and Flotillin-1 while also suppressing migration on the Stiff matrix. Together, cholesterol depletion and ITGB1 knockdown support a functional association between raft-associated membrane changes and stiffness-promoted wound closure. Because MβCD broadly depletes membrane cholesterol, however, its effects should be interpreted together with the GM1, raft-fractionation, and ITGB1-knockdown data rather than attributed exclusively to lipid raft disruption. Because conventional confocal imaging and detergent-based fractionation do not directly resolve nanoscale lipid raft domains, and membrane order was not independently assessed using probes such as Laurdan or di-4-ANEPPDHQ, our findings support changes in raft-associated membrane organization rather than direct demonstration of de novo lipid raft formation.

**Figure 7:**
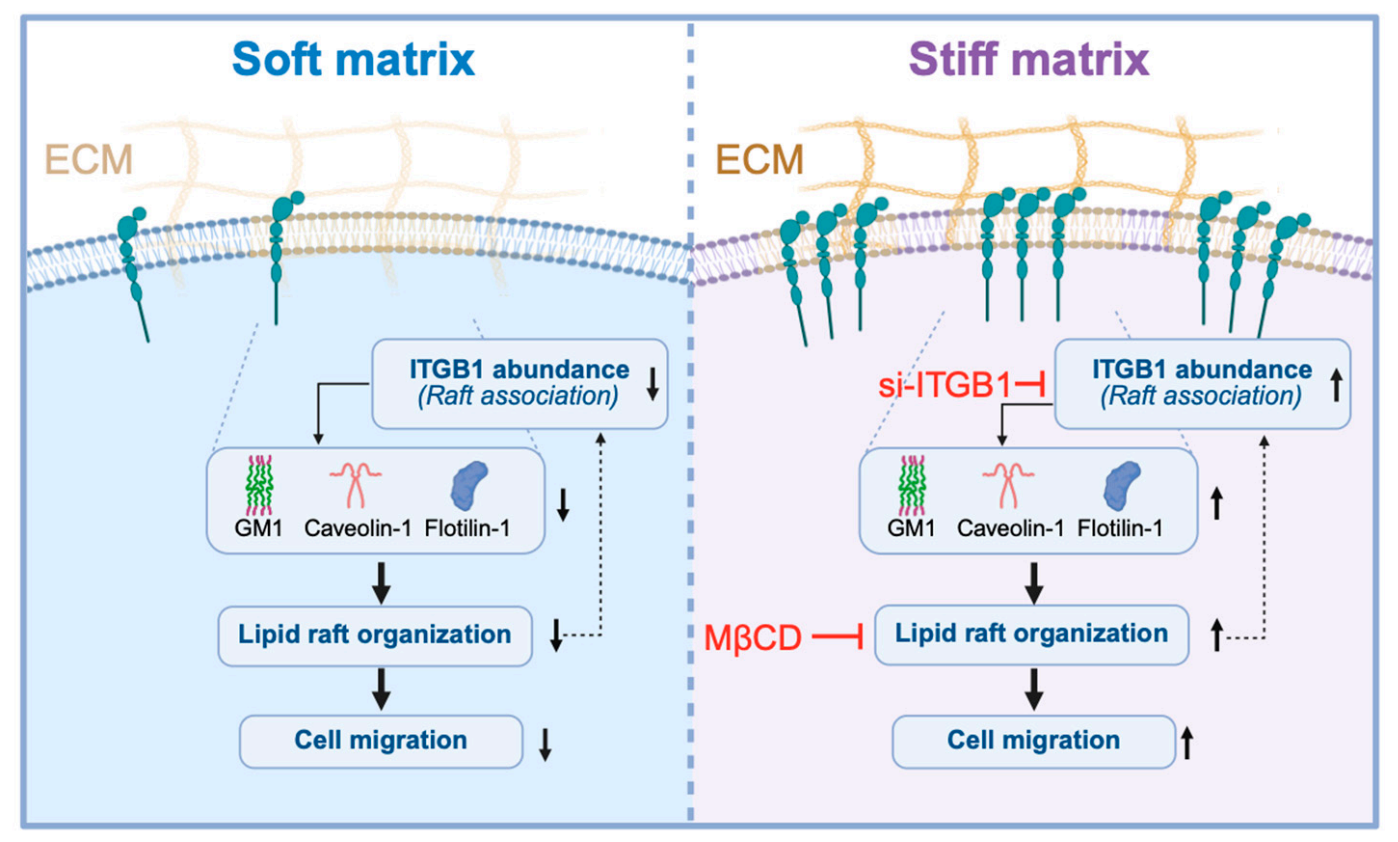
Proposed working model linking matrix stiffness, ITGB1, lipid raft–associated membrane organization, and HCC cell migration. Compared with the Soft matrix condition, the Stiff matrix is associated with increased ITGB1 abundance and raft association, enhanced raft-associated features including GM1, Caveolin-1, and Flotillin-1, increased lipid raft organization, and enhanced HCC cell migration. ITGB1 knockdown attenuates raft-associated changes, whereas MβCD-mediated cholesterol depletion disrupts lipid raft organization. The reciprocal relationship depicted between ITGB1 and lipid raft organization reflects the possibility of bidirectional regulation rather than a strictly linear pathway. Created in BioRender (web-based version). Zhang, X. (2026). https://BioRender.com/s1ht8mx (accessed on 10 September 2026).

Based on these findings, we propose a working model linking matrix stiffness, ITGB1 abundance, raft-associated membrane changes, and HCC cell migration (Figure 7). The present data support functional relationships among these events but do not establish their temporal sequence. In particular, whether changes in ITGB1 precede, occur concurrently with, or follow changes in lipid raft–associated membrane organization remains to be determined. Lipid rafts are dynamic membrane structures shaped by membrane lipids, proteins, and the cortical cytoskeleton and can regulate receptor partitioning and local signaling assembly [10,13,39,40,41].

## 4. Materials and Methods

### 4.1. Preparation and Mechanical Characterization of Polyacrylamide Hydrogels

Polyacrylamide (PA) hydrogels with two stiffness levels were prepared using a protocol previously established by our group [9]. Stock solutions of acrylamide (40%, *w*/*v*) and bis–acrylamide (2%, *w*/*v*) were obtained from Sigma–Aldrich (Sigma-Aldrich, St. Louis, MO, USA) and diluted with distilled water. Both hydrogel formulations contained 10% (*w*/*v*) acrylamide, with final bis–acrylamide concentrations of 0.03% (*w*/*v*) for the Soft hydrogels and 0.30% (*w*/*v*) for the Stiff hydrogels. Approximately 10 mL of each precursor solution were supplemented with 50 μL of 10% (*w*/*v*) ammonium persulfate (APS; Beyotime, Shanghai, China), and polymerization was initiated by adding 10 μL of N,N,N′,N′–tetramethylethylenediamine (TEMED; Macklin, Shanghai, China). After polymerization, the hydrogels were washed with distilled water to remove residual reagents.

For surface functionalization, the hydrogel surfaces were treated with 200 μg/mL sulfosuccinimidyl 6–(4′–azido–2′–nitrophenylamino) hexanoate (sulfo–SANPAH; Thermo Fisher Scientific, Waltham, MA, USA) and exposed to 365 nm UV light for 25 min. After washing, the activated hydrogels were incubated overnight at 37 °C with 0.1 mg/mL rat-tail collagen type I (COL1; Hangzhou Shengyou Biotechnology, Hangzhou, China). The mechanical properties of the PA hydrogels were directly characterized using the self–weighing tensile method as previously described [42]. Briefly, planar hydrogels prepared using the same formulations employed for cell culture were cut into rectangular strips, and the deformation of each hydrogel under a defined gravitational load was measured. Strain was calculated as the change in hydrogel length relative to its initial length (ΔL/L), and Young’s modulus was calculated from the applied stress and the resulting strain according to E = (G/A)/(ΔL/L), where G represents the applied gravitational load and A represents the cross-sectional area of the hydrogel sample. Six independently prepared hydrogels were mechanically characterized for each condition. The Young’s moduli of the Soft and Stiff hydrogels were 7.6 ± 0.22 kPa and 59.3 ± 1.47 kPa, respectively (mean ± SD, *n* = 6 independent hydrogels per group). The same hydrogel formulations and standardized preparation procedure were used throughout all subsequent matrix-stiffness experiments.

### 4.2. Cell Culture

The human HCC cell lines MHCC-97H and HCC-LM3 were obtained from the Liver Cancer Institute, Zhongshan Hospital, Fudan University (Shanghai, China). Cells were cultured in high-glucose Dulbecco’s modified Eagle’s medium (DMEM; Gibco, Thermo Fisher Scientific, Waltham, MA, USA) supplemented with 10% fetal bovine serum (FBS; HyClone, UT, USA), 2 mM glutamine (Solarbio, Beijing, China), 100 U/mL penicillin, and 100 μg/mL streptomycin (Solarbio Life Sciences, Beijing, China). Cells were maintained at 37 °C in a humidified incubator containing 5% CO_2_. At approximately 80–90% confluence, cells were detached with 0.25% trypsin–0.02% EDTA (Procell, Wuhan, China) and passaged.

For matrix stiffness experiments, unless otherwise specified, cells were seeded onto collagen I–coated Soft or Stiff PA hydrogels in six-well plates at approximately 5 × 10^5^ to 1 × 10^6^ cells per well and cultured for 48 h before subsequent analyses.

### 4.3. Wound-Healing Assay

Cell migration was assessed using a wound-healing assay with ibidi Culture–Inserts (ibidi, Gräfelfing, Germany). The inserts were placed on collagen I–coated Soft or Stiff PA hydrogels in 24-well plates. Cells were suspended at a density of approximately 1 × 10^6^ cells/mL, and 100 µL of the cell suspension were seeded into each chamber. After the cells reached confluence, the inserts were carefully removed to generate a defined cell-free gap. The cultures were washed twice to remove detached cells and debris, followed by the addition of serum-free medium. For experiments without MβCD or cholesterol treatment, images were acquired immediately after gap formation and designated as the 0 h time point. The same fields were imaged 12 h later. For MβCD and cholesterol-replenishment experiments, 0 h was defined after completion of the corresponding treatment and time-matched control incubation, as detailed below.

For MβCD wound-healing experiments, cells on the Stiff matrix were exposed to 5 mM MβCD for 60 min immediately after removal of the Culture–Inserts. During the same period, the Soft and Stiff control groups were incubated in serum-free medium without MβCD. After this 60 min period, all groups were washed with PBS and supplied with fresh serum-free medium. Images were then acquired and designated as the 0 h time point for all groups. The same fields were imaged 12 h later. MβCD was absent during this 12 h observation period.

For cholesterol-replenishment wound-healing experiments, cells were assigned to the Stiff, Stiff + MβCD, and Stiff + MβCD + cholesterol groups. Immediately after removal of the Culture–Inserts, the two MβCD groups received 5 mM MβCD for 60 min. The Stiff control group underwent a time-matched incubation in serum-free medium without MβCD. After PBS washing, the cholesterol replenishment group received an MβCD–cholesterol complex containing 125 µM cholesterol for 4 h. During this period, the other two groups underwent a time-matched incubation in serum-free medium without the MβCD–cholesterol complex. After this 4 h period, all groups were washed with PBS and supplied with fresh serum-free medium. Images were then acquired and designated as the 0 h time point for all groups. The same fields were imaged 12 h later.

For ITGB1 knockdown experiments, si-NC– or si-ITGB1–transfected cells were prepared as described in Section 4.11 and subsequently seeded into Culture–Inserts on Soft or Stiff PA hydrogels. After the cells reached confluence, the inserts were removed, and the cultures were processed and imaged at 0 and 12 h as described above.

Cell–free areas were quantified using ImageJ version 1.8.0 (National Institutes of Health, Bethesda, MD, USA). Wound closure was calculated from the change in the cell–free area between the experiment-specific 0 h baseline and 12 h. For MβCD and cholesterol replenishment experiments, the baseline was established after completion of the respective treatment and time-matched control incubation.

### 4.4. Reverse Transcription Quantitative PCR (RT-qPCR)

Total RNA was extracted using the SteadyPure Quick RNA Extraction Kit (Accurate Biology, Changsha, China; Cat. No. AG21023). Genomic DNA removal and reverse transcription were performed using ABScript Neo RT Master Mix for qPCR with gDNA Remover (ABclonal, Wuhan, China; Cat. No. RK20433) on a T100 Thermal Cycler (Bio–Rad Laboratories, Hercules, CA, USA). Quantitative PCR was performed using 2× Universal SYBR Green Fast qPCR Mix (ABclonal; Cat. No. RK21203) on a CFX96 Real-Time PCR Detection System (Bio–Rad Laboratories). ACTB was used as the reference gene, and relative mRNA expression was calculated using the 2^−ΔΔCt^ method after normalization to ACTB. Primer sequences are listed in Table A1.

### 4.5. Protein Extraction and Western Blotting

For total-cell protein analysis, cells were collected following the indicated treatments and lysed on ice in RIPA buffer (Beyotime, Shanghai, China) supplemented with 1% protease inhibitor cocktail (Beyotime, Shanghai, China) and 2% phosphatase inhibitor cocktail. Protein concentrations were determined using a BCA Protein Assay Kit (Beyotime, Shanghai, China). For analysis of raft-enriched and non-raft fractions, samples were prepared and quantified as described in Section 4.9.

Protein samples were mixed with 5× loading buffer and heated at 95 °C for 5 min. Equal amounts of protein (30 μg per lane) were separated by 10% SDS–PAGE using a PAGE Gel Fast Preparation Kit (EpiZyme, Shanghai, China; Cat. No. PG112) and transferred onto 0.45 μm PVDF membranes (Millipore, Billerica, MA, USA). Membranes were blocked with 5% skim milk in TBST for 1 h at room temperature and incubated overnight at 4 °C with primary antibodies against Flotillin-1 (1:10,000; Proteintech, Wuhan, China, Cat. No. 15571–1–AP), Caveolin-1 (1:10,000; Proteintech, Wuhan, China, Cat. No. 16447-1-AP), ITGB1 (1:10,000; Proteintech, Wuhan, China, Cat. No. 12594-1-AP), or β-actin (1:20,000; ABclonal, Wuhan, China, Cat. No. AC004), diluted in Primary Antibody Dilution Buffer (Beyotime, Shanghai, China).

After washing with TBST, membranes were incubated for 1 h at room temperature with HRP-conjugated goat anti-rabbit IgG (1:10,000; Solarbio, Beijing, China; Cat. No. SE134) or goat anti-mouse IgG (1:10,000; Solarbio; Cat. No. SE131), as appropriate. Immunoreactive bands were detected using enhanced chemiluminescence reagent (Yeasen, Shanghai, China; Cat. No. 36208ES60), and chemiluminescent signals were acquired using a Bio–OI imaging system (Guangzhou, China).

For densitometric analysis, target-protein signals in total-cell lysates (Total) and detergent-soluble non-raft fractions (Non-rafts) were normalized to β-actin. For raft-enriched fractions (Rafts), PVDF membranes were stained with Ponceau S immediately after protein transfer (Beyotime; Shanghai, China, Cat. No. P0022–120ml) and imaged before blocking. Target-protein signals were normalized to the integrated Ponceau S total-protein signal of the corresponding lane. Band intensities and Ponceau S signals were quantified using ImageJ version 1.8.0 (National Institutes of Health, Bethesda, MD, USA).

### 4.6. GM1 Labeling and ITGB1 Immunofluorescence Staining

Cell-surface ganglioside GM1 was labeled with CF^®^488A-conjugated cholera toxin B subunit (CTB; Baotian Biotechnology, Shanghai, China; Cat. No. 90-2615) following culture on Soft or Stiff PA hydrogels and the indicated treatments. Cells were rinsed with prechilled Hanks’ balanced salt solution (HBSS; Beyotime, Shanghai, China; Cat. No. C0218) containing 0.5% BSA and then incubated with 10 μg/mL CF^®^488A–CTB diluted in the same buffer for 30 min at 4 °C in the dark. After washing with prechilled HBSS containing 0.5% BSA, cells were fixed with 4% paraformaldehyde for 15 min at 4 °C. This labeling procedure was used for both GM1-only and ITGB1–GM1 double-labeling experiments.

For GM1–only experiments, fixed cells were washed with PBS and counterstained with DAPI before fluorescence imaging. For ITGB1–GM1 double labeling, fixed cells were processed without permeabilization. After washing with PBS, cells were blocked with 1% BSA in PBS for 1 h at room temperature and incubated overnight at 4 °C with anti-ITGB1 antibody (1:200; Proteintech, Wuhan, China; Cat. No. 12594-1-AP). Cells were then washed with PBS and incubated for 1 h at room temperature in the dark with Cy3-conjugated goat anti-rabbit IgG (H+L) secondary antibody (1:200; Proteintech, Wuhan, China; Cat. No. SA00009-2). Nuclei were counterstained for 10 min with ready-to-use DAPI solution (10 μg/mL; Solarbio, Beijing, China; Cat. No. C0065).

Fluorescence images were acquired using an LSM 900 confocal laser-scanning microscope (Carl Zeiss, Germany), with acquisition settings kept constant across groups within the same experiment. Area-normalized GM1 fluorescence was quantified using ImageJ version 1.8.0 (National Institutes of Health, Bethesda, MD, USA). For cells with clearly discernible contours, whole-cell regions of interest were manually delineated using identical criteria across experimental groups. Background fluorescence was determined from cell-free regions within the corresponding image. For each cell, background-corrected integrated GM1 fluorescence was calculated as integrated density (projected cell area × mean background intensity) and subsequently normalized to the projected cell area. Values from individual cells were averaged within each independent experiment for statistical analysis. ITGB1–GM1 colocalization was analyzed using ZEN 3.8 software (Carl Zeiss Microscopy GmbH). Intensity thresholds for the two fluorescence channels were determined using the Costes automatic thresholding method. Pixels were classified as channel 1–only, channel 2–only, colocalized, or background according to the two-channel intensity scatter plot. The colocalization rate was calculated as the proportion of colocalized pixels among total foreground pixels after background exclusion: colocalization rate (%) = Q3/(Q1 + Q2 + Q3) × 100.

### 4.7. MβCD and Cholesterol Treatment

Methyl-β–cyclodextrin (MβCD; Macklin, Shanghai, China; Cat. No. M830139) was prepared in serum-free medium. Cells cultured on the Stiff matrix were treated with 5 mM MβCD for 60 min at 37 °C, whereas the corresponding Stiff controls were incubated in serum-free medium without MβCD under otherwise identical conditions. The 60 min acute cholesterol-depletion period was selected with reference to previous MβCD studies in HCC cells [38].

For cholesterol–replenishment experiments, cholesterol (Solarbio, Beijing, China; Cat. No. C8280) was complexed with MβCD at an 8:1 molar ratio of MβCD to cholesterol, with reference to a previously reported method [34]. Following MβCD-mediated cholesterol depletion, cells were washed with PBS and incubated with the MβCD–cholesterol complex at a final cholesterol concentration of 125 μM for 4 h at 37 °C. After cholesterol replenishment, cells were processed for GM1 labeling or wound-healing analysis as described in Section 4.6 and Section 4.3, respectively.

For GM1 analysis, cells were cultured on Stiff PA hydrogels for 48 h before MβCD treatment. After the 60 min exposure, MβCD was removed by washing with PBS, and cells were subsequently processed for GM1 labeling as described in Section 4.6.

For wound-healing experiments, treatment schedules and time-matched control incubations are described in Section 4.3. In all groups, 0 h was defined after completion of the relevant treatment or control incubation and subsequent medium replacement.

### 4.8. Cell Viability Assay

MHCC-97H and HCC-LM3 cells were seeded at 1.5 × 10^4^ cells per well in 24-well plates. For the Control group, cells were cultured directly on standard tissue–culture-treated plates without PA hydrogels, whereas cells in the Soft, Stiff, and Stiff + MβCD groups were seeded onto the corresponding PA hydrogels. After 24 h, cells in the Stiff + MβCD group were treated with 5 mM MβCD for 60 min at 37 °C. The Control, Soft, and Stiff groups were incubated in serum-free medium without MβCD under otherwise identical conditions.

After treatment, the cultures were washed with PBS, and 500 μL of complete medium containing 50 μL of Cell Counting Kit–8 reagent (CCK-8; Meilunbio, Dalian, China; Cat. No. MA0218) were added to each well. Following incubation for 2 h at 37 °C, a 100 μL aliquot from each well was transferred to a 96–well plate, and absorbance was measured at 450 nm using a microplate reader. Cell viability (%) was calculated by normalizing the absorbance of each group to the mean absorbance of the Control group, which was defined as 100%.

### 4.9. Lipid Raft Isolation

Raft-enriched fractions were isolated using the ULTRARIPA^®^ Kit for Lipid Raft (BioDynamics Laboratory Inc., Tokyo, Japan; Cat. No. F015) with modifications to the manufacturer’s instructions. After culture on Soft or Stiff PA hydrogels and the indicated treatments, cells were washed with PBS, harvested, and pelleted. Cell pellets were lysed in 100–200 μL of ice–cold A buffer (RIPA buffer), with the volume adjusted according to pellet size. Samples were disrupted by repeated pipetting and sonication and incubated on ice for 10 min. The lysates were then centrifuged at 12,000× *g* for 10 min at 4 °C, and the resulting supernatants were collected as detergent-soluble non-raft fractions, hereafter referred to as Non-rafts.

The remaining detergent-resistant pellets were washed with 0.5 mL of ice-cold A buffer, resuspended by repeated pipetting or vortexing, and centrifuged again at 12,000× *g* for 10 min at 4 °C. After removal of the wash supernatants, the pellets were resuspended in 50–100 μL of B buffer equilibrated to room temperature, mixed thoroughly, and incubated for 5 min at room temperature. Samples were then centrifuged at 12,000× *g* for 10 min at 25 °C, and the resulting supernatants were collected as detergent-resistant, raft-enriched fractions, hereafter referred to as Rafts.

Protein concentrations of Rafts and Non-rafts were determined using a BCA Protein Assay Kit (Yeasen, Shanghai, China; Cat. No. 20201ES76), and equal amounts of protein were subjected to Western blotting as described in Section 4.5. Total samples were prepared separately from whole-cell lysates as described in Section 4.5.

### 4.10. Cholesterol Quantification

Total cholesterol (TC) levels were quantified using a Total Cholesterol Content Assay Kit (Solarbio, Beijing, China; Cat. No. BC1985) according to the manufacturer’s instructions. For total cellular cholesterol measurement, cells cultured on Soft or Stiff PA hydrogels for 48 h were harvested and resuspended in the extraction solution provided with the kit. Cell suspensions were disrupted by sonication on ice and centrifuged at 10,000× *g* for 10 min at 4 °C, and the resulting supernatants were collected for cholesterol determination. For measurement of cholesterol in raft-enriched membrane fractions, the raft-enriched fractions isolated as described in Section 4.9 were directly subjected to the same cholesterol assay.

For the colorimetric reaction, 10 μL of each sample were mixed with 500 μL of freshly prepared working solution and incubated at 37 °C for 30 min. After incubation, 200 μL of the reaction mixture were transferred to a 96-well plate, and absorbance was measured at 500 nm using a microplate reader. Cholesterol concentrations were calculated from a cholesterol standard curve according to the manufacturer’s instructions. Total cellular cholesterol and cholesterol in the raft-enriched fractions were normalized to the corresponding cell number and expressed as μmol/10^6^ cells.

### 4.11. siRNA Transfection

An siRNA duplex targeting ITGB1 (si-ITGB1) and a negative–control siRNA (si-NC) were synthesized by Tsingke Biotechnology Co. (Beijing, China). The sequences were identical to those used in our previous study [9] and are listed in Table A2. Cells were transfected with siRNA at a final concentration of 50 nM using RNAiBoost Transfection Reagent (Yeasen, Shanghai, China) according to the manufacturer’s instructions. ITGB1 knockdown efficiency was verified by Western blotting 36 h after transfection.

For matrix stiffness experiments, parallel siRNA–transfected cells were trypsinized 36 h after transfection and seeded onto collagen I-coated Soft or Stiff PA hydrogels. According to matrix stiffness and siRNA treatment, cells were assigned to four groups: Soft + si-NC, Soft + si-ITGB1, Stiff + si-NC, and Stiff + si-ITGB1. Unless otherwise specified, cells were cultured on the hydrogels for an additional 48 h before subsequent analyses.

### 4.12. Statistical Analysis

Statistical analyses and graph preparation were performed using GraphPad Prism 9.5.1 (GraphPad Software Inc., San Diego, CA, USA). Data are presented as mean ± standard deviation (SD). Unless otherwise specified, data were obtained from three independent experiments. Comparisons between two groups were performed using two-tailed unpaired Student’s t-tests. For datasets containing three or more groups, one-way ANOVA followed by Tukey’s multiple-comparisons test was used. For experiments involving two independent factors, matrix stiffness and siRNA treatment, two-way ANOVA followed by Tukey’s multiple-comparisons test was used. A *p*-value < 0.05 was considered statistically significant.

## 5. Conclusions

Using two HCC cell lines cultured on stiffness-defined PA hydrogels, we found that matrix stiffening enhanced HCC cell migration and was associated with increased lipid raft–associated membrane organization, as indicated by increased Area-normalized GM1 fluorescence and elevated Caveolin-1 and Flotillin-1 expression. Stiff matrix also increased total ITGB1 abundance, ITGB1–GM1 colocalization, and ITGB1 levels in raft-enriched fractions. MβCD-mediated membrane cholesterol depletion reduced GM1 fluorescence and migration, whereas ITGB1 knockdown decreased GM1 fluorescence and Caveolin-1 and Flotillin-1 levels in raft-enriched fractions while suppressing migration under Stiff-matrix conditions. These findings support a contributory role for ITGB1 in stiffness-associated lipid raft organization and identify lipid raft-associated membrane organization as a membrane-level process linked to stiffness-promoted HCC cell migration.

## Data Availability

The data presented in this study are available from the corresponding author upon reasonable request.

## Abbreviations

The following abbreviations are used in this manuscript:

ANOVA	Analysis of variance
APS	Ammonium persulfate
BCA	Bicinchoninic acid
BSA	Bovine serum albumin
COL1	Collagen type I
CTB	Cholera toxin B subunit
DAPI	4′,6–diamidino–2–phenylindole
DMEM	Dulbecco’s modified Eagle’s medium
ECM	Extracellular matrix
EDTA	Ethylenediaminetetraacetic acid
FBS	Fetal bovine serum
GM1	Ganglioside GM1
HBSS	Hanks’ balanced salt solution
HCC	Hepatocellular carcinoma
ITGB1	Integrin β1
MβCD	Methyl-β–cyclodextrin
PA	Polyacrylamide
PBS	Phosphate–buffered saline
PVDF	Polyvinylidene fluoride
RIPA	Radioimmunoprecipitation assay
RT-qPCR	Reverse transcription quantitative polymerase chain reaction
TBST	Tris–buffered saline containing Tween 20
TEMED	N,N,N′,N′–Tetramethylethylenediamine
UV	Ultraviolet

## Appendix A

### Appendix A.1. RT-qPCR Primer Sequences

Primer sequences applied in this study for RT-qPCR are listed in Table A1.

**Table A1:** RT-qPCR primer sequences.

	Forward Primer (5′–3′)	Reverse Primer (5′–3′)
*FLOT1*	AAGCTGCCCCAGGTGGCAGAGG	TGTTCTCAAAGGCTTGTGATTCACC
*CAV1*	ACGTGGTCAAGATTGACTTTGAAG	AGATGTGCAGGAAAGAGAGAATG
*ACTB*	CATGTACGTTGCTATCCAGGC	CTCCTTAATGTCACGCACGAT

### Appendix A.2. siRNA Sequences

siRNA sequences of ITGB1 are listed in Table A2.

**Table A2:** siRNA sequences.

	Sense (5′–3′)	Antisense (5′–3′)
si-ITGB1	CCACAGCAGUUGGUUUUGCTT	GCAAAACCAACUGCUGUGGTT
si-NC	UUCUCCGAACGUGUCACGUTT	ACGUGACACGUUCGGAGAATT
